# Optimizing topical glaucoma treatment outcomes: When less is better. A review on the clinical benefits of reducing treatment burden in glaucoma management

**DOI:** 10.1177/11206721261438585

**Published:** 2026-04-17

**Authors:** Ronald Marinus Petrus Cornelis de Crom, Luis Abegão Pinto, Ingeborg Stalmans, Andrew Tatham, Verena Prokosch, Miriam Kolko, Jose Manuel Benitez-del-Castillo

**Affiliations:** 1 University Eye Clinic Maastricht, 199236 Maastricht University Medical Centre+, Maastricht, The Netherlands; 2 Department of Ophthalmology. ULS Santa Maria, Lisbon, Portugal; 3 Faculty of Medicine of Lisbon University, Lisbon, Portugal 6. Princess Alexandra Eye Pavilion and University of Edinburgh, Edinburgh, UK; 4 Department of Ophthalmology, 26657 University Hospitals UZ Leuven, Leuven, Belgium; 5 Research Group Ophthalmology, Department of Neurosciences, Catholic University KU Leuven, Leuven, Belgium; 6 Princess Alexandra Eye Pavilion and University of Edinburgh, UK; 7 University Eye Hospital Cologne, Cologne, Germany; 8 Department of Drug Design and Pharmacology, University of Copenhagen; Department of Ophthalmology; Copenhagen University Hospital; Department of Ophthalmology, 4321 Aalborg University Hospital; Eye hospital Your Eye Doctors (Dine Øjenlæger), Sundhedshuset, Rungsted Kyst, Danemark; 9 Hospital Clinico San Carlos, 16267 Universidad Complutense, Ocumed-Clinica Rementeria, Madrid, Spain

**Keywords:** glaucoma, preservative-free, low concentration, fixed combination, ocular hypertension, adherence, ocular surface, sustainability

## Abstract

Glaucoma, a leading cause of irreversible blindness, is primarily managed by lowering intraocular pressure, yet treatment remains limited by poor adherence, ocular surface toxicity, and systemic adverse effects. This review focuses specifically on treatment simplification strategies in topical glaucoma therapy, exploring the paradigm shift towards a “Less is better” approach in glaucoma care, with particular emphasis on the use of preservative -free formulations, lower active ingredient concentrations, and fixed combinations. These approaches aim to improve treatment tolerability, adherence, and clinical outcomes while minimizing toxicity, complexity, and environmental burden. Although barriers such as higher costs and limited long-term data remain, this new approach represents a patient-centred, sustainable, and clinically effective model for the future of glaucoma management.

## Introduction

Glaucoma is characterized by a progressive loss of retinal ganglion cells and is the second leading cause of the global blindness in people aged over 50 years old in 2020.^1^ Approximately 76–80 million people are currently living with this disease globally, with 12.3 million cases in Europe.^2^ Glaucoma is projected to affect approximately 111.8 million people worldwide by 2040.^3^ Elevated intraocular pressure (IOP) is a significant risk factor for glaucoma progression, and lowering IOP is to date the only proven way to slow disease progression.^4^ Treatment options to reduce IOP include eye drops, laser procedures, and surgical interventions.^5,6^ The first-line medical approach typically involves topical treatment monotherapy, but a significant proportion of patients require a combination of two or more medications to achieve target IOP and prevent or delay optic nerve damage. Prostaglandin analogues, the most effective IOP-lowering agents,^7^ are often combined with other drug classes such as β-blockers, carbonic anhydrase inhibitors, rho-kinase inhibitors, or α₂-adrenergic agonists.^8^

Long-term success in glaucoma therapy depends on both treatment efficacy and patient adherence. However, adherence was shown to be low in glaucoma patients,^9^ often due to adverse effects (AEs) or complex drug regimen associated with conventional therapies.^10^ Ocular surface disease (OSD) is more common in individuals with glaucoma than in the general population, making treatment tolerance a key factor when choosing suitable IOP-lowering medications.^11^ Recent evidence indicates that a higher number of topical medications worsen meibomian gland obstruction severity, further contributing to ocular surface compromise and discomfort.^12^ Evidence showed that poor glaucoma treatment adherence significantly accelerated visual field loss,^13^ highlighting the importance of consistent treatment to prevent disease progression. The prolonged use of preserved formulations, especially those with benzalkonium chloride (BAK), has been linked to eye surface toxicity, inflammation, and discomfort, which can negatively impact patient adherence.^14,15^ Increasing studies have highlighted better adherence with preservative-free (PF) formulations compared to preserved ones.^16,17^ Additionally, the complexity of using multiple medications and the risk of systemic absorption of active ingredients can cause AEs and treatment fatigue, further compromising patient adherence and the success of therapy.^18^

In response to these challenges of long-term topical therapy, a shift toward treatment simplification approaches consisting in simplified regimens has gained traction in ophthalmology leading to the concept of “Less is better”. This strategy simplifies treatment to enhance adherence, tolerance, and long-term outcomes.^19,20^ Key measures include PF formulations, lower drug concentrations, fixed combinations, or reducing the number of instillations with fixed combinations or new innovative formulations to decrease ocular toxicity and treatment burden without compromising IOP control. Focusing on topical treatments, this review examines how these elements can affect the efficacy, tolerance, quality of life, and environmental sustainability of topical glaucoma therapies.

## Methods

A narrative literature review was conducted in February 2025 to summarize current evidence on treatment simplification strategies in topical glaucoma therapy. PubMed searches combined disease terms (*glaucoma*), preservatives (preservat* OR BAK OR benzalkonium OR phosphate*), and treatment or dose strategies (monotherapy OR combination OR switch* OR “fixed combination” OR “low dose” OR dose reduc* OR “lower dose” OR “lowering dose”). Embase searches used Emtree and free-text terms for disease (‘glaucoma’/mj OR ‘chronic glaucoma’), dose modification (‘drug dose reduction’/exp OR ‘dosage decrease’ OR ‘dose reduction’ OR ‘drug tapering’), preservatives (‘preservative’/exp OR ‘phosphate’/exp), and combination therapy (‘fixed combination’). Only human studies reporting clinical trials, observational studies, guidelines, or systematic reviews were included, with no date restrictions applied. A total of 1,114 articles were retrieved. Relevant articles were screened by two independent reviewers, and studies addressing clinical efficacy, safety, adherence, or environmental impact of simplified glaucoma therapy were included. Exclusion criteria were non-topical interventions, studies lacking clinical or adherence outcomes, or insufficient methodological detail.

## Clinical issues: Limitations of current treatments

**Figure 1.**
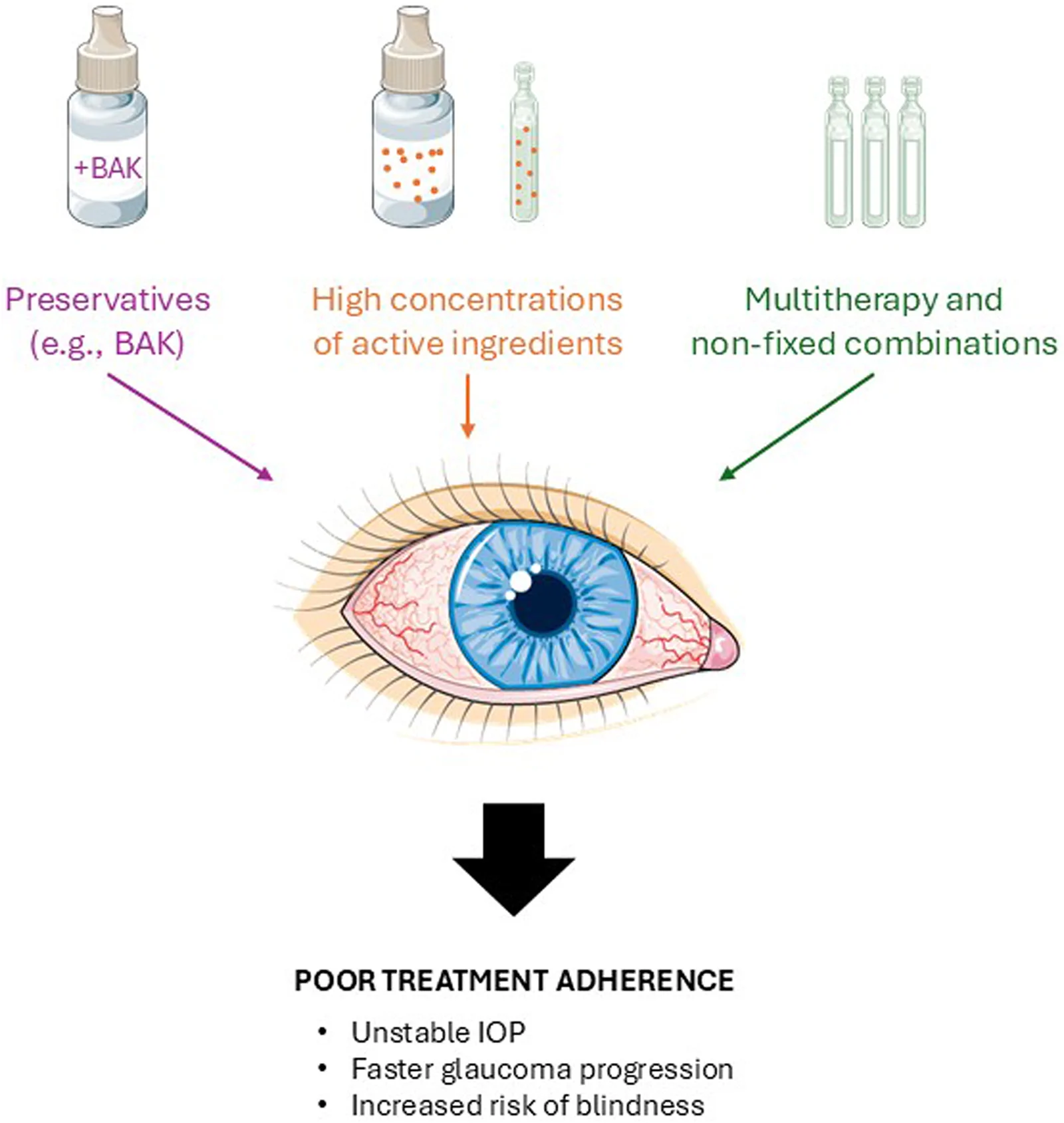
Overview of clinical limitations in current glaucoma therapies.

BAK: benzalkonium chloride; IOP: intraocular pressure. Figure partly designed with Smart servier, https://smart.servier.com/

Despite advances in glaucoma therapy, important limitations persist. These include preservative-related ocular toxicity, AEs related to active ingredients, and burden of complex multi-drug regimens (Figure 1).

**Figure 2.**
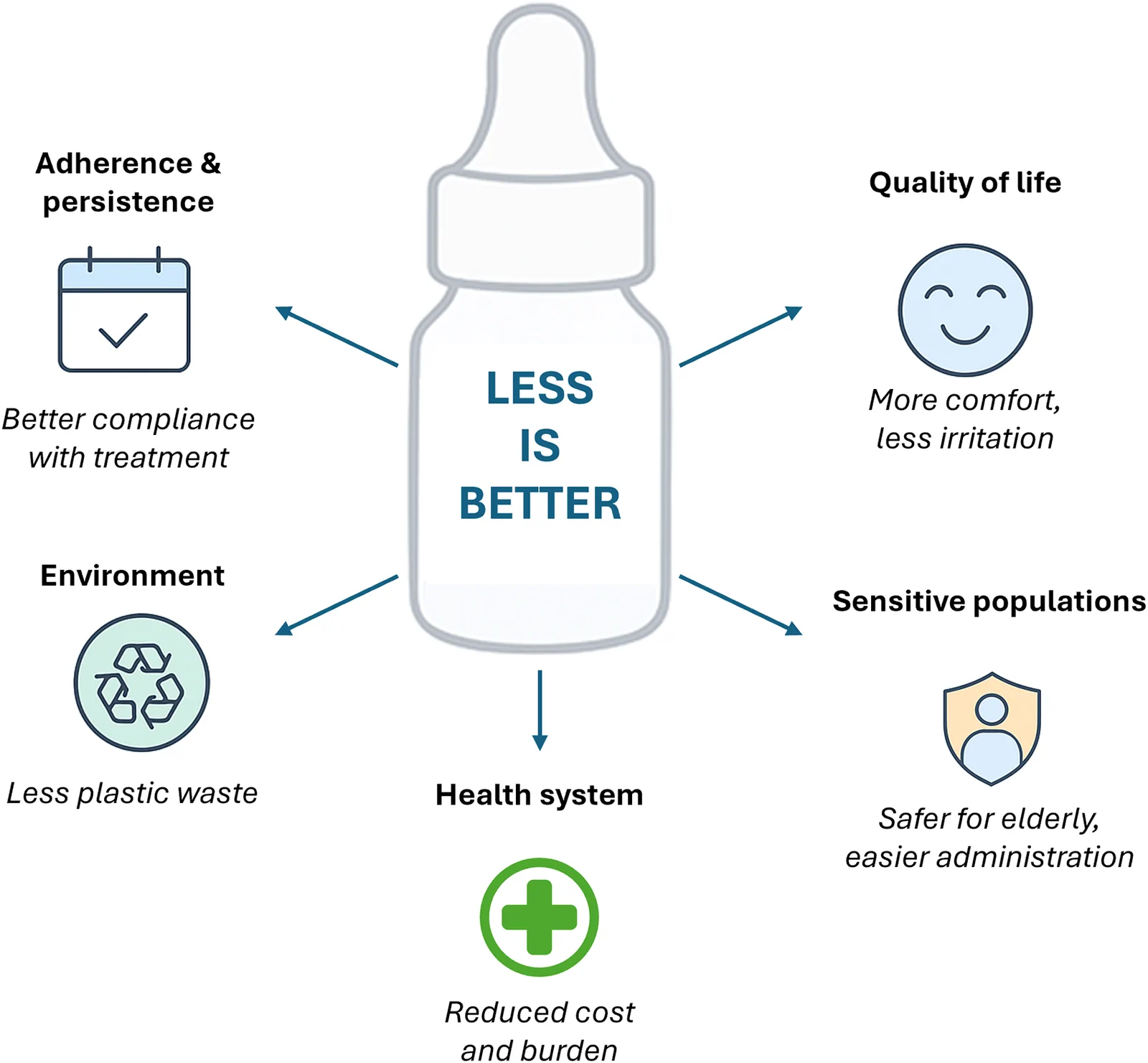
Treatment simplification approaches in glaucoma management: scientific rationale and global impact.

### Toxicity of preservatives

#### Impact on the ocular surface and deeper tissues

In glaucoma therapy, the chronic use of preservatives, particularly BAK, has been extensively linked to ocular surface toxicity, chronic inflammation, and disruption of homeostasis.^21–23^ BAK can damage the corneal and conjunctival epithelium, impair tear film stability, and reduce goblet cell function, which may decrease treatment adherence.^24–28^ These effects are especially pronounced in patients receiving multiple preserved medications, resulting in reduced treatment adherence and increased use of artificial tears.^20,29^ Recent findings also suggest that BAK may disrupt the ocular surface microbiota, contributing to further instability and inflammation.^30–33^ Beyond the ocular surface, BAK has been shown to penetrate deeper tissues and contribute to trabecular meshwork degeneration by increasing matrix metalloproteinase-9 activity and activating Müller glial cells.^34–38^ Taken together, these processes trigger inflammatory cascades that may impair aqueous outflow, compromise IOP regulation, and potentially accelerate glaucoma progression.^39–42^

#### Increased surgical risk and risk of surgical failure

Studies have shown that long-term exposure to preserved medications significantly increased the risk of surgical failure in glaucoma patients, likely due to ocular surface toxicity and reduced medication tolerance.^25,43^ Indeed, in a nationwide cohort of 664,494 patients followed for a median of 4.8 years, the group using preserved eyedrops had more than a fourfold increased risk of undergoing glaucoma surgery compared to patients using PF drops, while patients using a mix of both had almost a twofold increased risk.^44^

On the other hand, preoperative exposure to BAK-preserved medications has been associated with a higher risk of trabeculectomy failure.^45^ Additionally, another study showed that eyes with higher ocular surface inflammation scores showed shorter postoperative survival and worse outcomes (p ≤ 0.03).^46^ This increased failure rate is likely due to chronic subclinical inflammation and conjunctival remodelling, resulting in fibroblast activation and subconjunctival fibrosis in eyes previously exposed to preservatives.^47,48^

### Adverse effects linked to the active molecule

#### Ocular adverse effects

The primary goal of glaucoma treatment is to preserve the patient's visual function and quality of life at a sustainable cost, balancing both therapeutic efficacy and the burden of treatment.^11^ IOP-lowering drugs currently available include prostaglandin analogues/prostamides, β-adrenergic receptor antagonists, carbonic anhydrase inhibitors, α-adrenergic receptor agonists, and Rho kinase inhibitors^5,11^ (Table 1). While effective at lowering IOP, these drugs present a spectrum of ocular AEs described in Table 1,^11,18,49,51^ especially in patients using multiple medications over the long term.^52^ Generally well tolerated, prostaglandin analogues/prostamides can cause local side effects including conjunctival hyperaemia, burning or stinging, and increased pigmentation of the iris and periocular skin.^11^

**Table 1.** IOP-lowering drugs used in glaucoma and corresponding adverse effects.

Drug category	Mechanism of action	Adverse effects	Reference
Prostaglandin analogues / Prostamides	Increase uveoscleral outflow	Ocular: conjunctival hyperaemia, burning stinging, foreign body sensation, itching, Minimal systemic effects	^11,15,49,50^
β-adrenergic receptor antiagonists	Decrease aqueous humour production	Ocular: minimal ocular effects Systemic: cardiovascular and respiratory risks	^11,15,49–51^
Carbonic anhydrase inhibitors (topical and oral)	Decrease aqueous humour production	Ocular: burning, visual disturbances Systemic (oral): fatigue, depression, electrolyte imbalances, rare severe reactions	^11,15,49,50^
α-adrenergic receptor agonists	Decrease aqueous humour production and increase uveoscleral outflow	Ocular: burning, allergic conjunctivitis Systemic: sedation, dry mouth, hypotension	^11,15,49,50^
Rho kinase inhibitors	Increase trabecular outflow	Ocular: conjunctival hyperaemia, congestion, and haemorrhage Generally well tolerated systemically	^11,15,49^
Parasympathomimetics / Cholinergic agents	Increase trabecular outflow via ciliary muscle contraction	Ocular: miosis, ciliary spasm, brow ache, night vision issues Systemic: GI disturbances, CNS effects, hypotension	^11,15,49^

#### Systemic adverse effects

Systemic risks from IOP-lowering medications can be important, particularly with long-term use and high active ingredient concentrations (Table 1).^50^ These risks vary by drug class and must be considered, especially in patients with comorbidities. Overall, it is now well established that patients who experienced AEs reported higher levels of non-adherence than those who did not (37.6% versus 18.4%; *p* = 0.004).^53^ Since poor treatment adherence leads to poor IOP control,^54^ the lowest effective concentration providing the target therapeutic effect while minimizing AEs should be used.^49^

### Problems related to dosing regimens

#### Difficulty of adherence

According to the European Glaucoma Society Guidelines, glaucoma treatment typically starts with monotherapy. If it does not effectively lower IOP or is not well tolerated, another monotherapy or a combination therapy can be considered.^11^ A real-world study in Portugal found that, despite the availability of fixed combinations, a significant portion of patients with glaucoma continued to be prescribed separate medications, resulting in unnecessarily complex regimens.^55,56^ Indeed, nearly one-third of patients on dual therapy were using two individual eye drops, and almost 30% were administering three or more drops daily. Since fixed combinations combining a prostaglandin analogue and a β-adrenergic receptor antagonist require only a single daily drop, their broader use could have immediately reduced drop burden in over 10% of patients.^56^ These findings underscore how optimizing treatment with fixed combinations can meaningfully reduce the number of daily administrations. Using multiple separate medications has been associated with poor adherence due to increased treatment complexity, frequency of dosing, and cumulative AEs, particularly when preserved formulations were involved.^57^ Several studies reported higher rates of missed doses and treatment discontinuation with non-fixed regimens.^58–60^ Finally, a patient-reported non-adherence study showed that for approximately one-third of patients, nonadherence with glaucoma therapy was a significant obstacle to therapeutic success.^53^

#### Influence on the efficacy and progression of glaucoma

Poor adherence and inconsistent medication use, particularly with complex or burdensome regimens, can lead to suboptimal IOP control. Clinical studies have consistently shown that effective IOP reduction remains the only proven strategy to delay disease progression and preserve visual function.^61–64^ When monotherapy fails to achieve adequate IOP control, persistent elevated IOP may contribute to progressive retinal ganglion cell loss, leading to visual field deterioration, impaired quality of life, and, eventually irreversible blindness.^62,65^ Therefore, early recognition of treatment insufficiency and simplification of complex regimens, such as switching to fixed combinations, can play a critical role in improving adherence, especially since good adherence during the first year predicts continued long-term adherence, enhancing tolerability, achieving stable IOP, and preventing long-term visual impairment.^66^

## Changing the paradigm: Towards a treatment simplification approach

Given the challenges faced by both ophthalmologists and patients in managing glaucoma, including preservative toxicity, AEs from higher concentration of active ingredients, and the frequent need for multiple medications, simplified approaches have become increasingly relevant to improve glaucoma care (Figure 2).

### Scientific foundations

#### Preservative-free versus preserved drugs

It is now well established that PF glaucoma medications can provide IOP reduction comparable to preserved formulations, while offering significantly improved ocular surface tolerance.^67^ Clinical studies have consistently demonstrated that PF prostaglandin analogues, such as latanoprost,^68–70^ bimatoprost 0.03% solution,^71^ bimatoprost 0.01% gel,^72,73^ travoprost,^74^ and tafluprost,^75,76^ are non-inferior in lowering IOP compared to their preserved counterparts. This was also the case of PF versus preserved β-adrenergic receptor antagonists,^77^ which led to fewer changes on ocular surface than preserved treatments.^78^ These efficacy data were associated with improved tolerability in terms of conjunctival hyperaemia and better ocular surface outcomes.^20,73,79–81^ Moreover, switching from the preserved to the PF prostaglandin analogue improved tear film stability and tear film breakup time, and reduced corneal staining and conjunctival hyperaemia.^82–84^ Interestingly, even preservative-optimized formulations, such as sofZia, Polyquad, or formulations containing less toxic preservatives, may represent transitional options when PF alternatives are unavailable or cost-prohibitive.^21,23,85^

#### Concentration of active ingredients

The evolution toward lower concentrations in ocular hypotensive treatments marks a major advance in glaucoma care, driven by the need to maintain efficacy while improving treatment tolerability. This shift began in the early 2000s with timolol 0.1% gel, the first low-concentration timolol gel formulation, allowing a prolonged residence time onto the ocular surface. It achieved a similar IOP-lowering effect as timolol 0.5% aqueous solution while significantly reducing systemic side effects and enabling a simplified dosing regimen from twice daily to once daily.^86–92^

Bimatoprost 0.03% was reformulated to improve tolerability, first as a preserved 0.01% formulation, and later as a 0.03% PF formulation, both of which displayed non-inferior efficacy while reducing conjunctival hyperaemia and ocular discomfort.^93–98^ The next step was the development of bimatoprost 0.01% PF gel, combining lower bimatoprost concentration with an innovative gel base.^73^ This gel differs from the previous one because it relies on the patented FDGt^®^—a fluid and gel technology combining three key ingredients: carbomer to enhance viscosity, sodium acetate trihydrate as viscosity-adjusting agent, and macrogol to improve solubility.^99^ Compared to preserved bimatoprost 0.01%, bimatoprost 0.01% PF gel showed a lower incidence and less worsening of conjunctival hyperaemia at both week 6 (20.1% vs. 29.3%) and week 12 (18.3% vs. 30.4%), with non-inferior efficacy.^84^ A phase III study conducted in the USA further confirmed these results. Indeed, bimatoprost 0.01% PF gel was noninferior to preserved bimatoprost 0.01% in IOP-lowering over 12 weeks, with fewer ocular AEs (28.1% vs. 34.8%) and less conjunctival hyperemia at Week 12 (54.8% vs. 63.2%) compared to preserved bimatoprost 0.01%.^100^ Pharmacokinetic data confirm that the gel prolongs ocular contact time, enabling effective IOP control at lower concentrations.^83^ Indeed, preclinical results demonstrated that PF bimatoprost 0.01% gel showed higher ocular exposure compared to preserved bimatoprost 0.01% (C_max_: 50.2 versus 26.3 ng/mL; AUC_0.5−8h_: 134.0 versus 67.0 ng·h/mL) and similar to preserved bimatoprost 0.03% (C_max_: 59.9 ng/mL; AUC_0.5−8h_: 148.0 ng·h/mL). In healthy humans, systemic exposure to bimatoprost was lower for the PF 0.01% gel (0.5248 and 0.5645 ng·min/mL on Days 1 and 15) than for the preserved 0.01% formulation (0.8461 and 0.7551 ng·min/mL), with no systemic accumulation observed in either group.^83^ This further highlights that lowering treatment concentration may improve tolerability and subsequent adherence to medication. Taken together, these advances underscore the role of innovative formulations (i.e., gel formulations) in achieving low-concentration efficacy with better tolerability, thereby addressing key needs in glaucoma management.

The first fixed combination of bimatoprost 0.03% and timolol 0.5% was initially launched in 2006 as a multi-dose formulation and later as a PF single-dose version. More recently, PF fixed combination of bimatoprost 0.01% and timolol 0.1% has emerged as the first PF low concentration fixed combination therapy incorporating gel formulation technology. Phase II clinical pharmacokinetic results demonstrated significantly lower systemic concentration of timolol after 12 weeks with PF bimatoprost 0.01%/timolol 0.1% gel compared to other combinations containing higher timolol concentrations.^101^ Regarding safety, treatment-related ocular AEs were generally mild to moderate, occurring in 13.8% of patients treated with PF bimatoprost 0.01%/timolol 0.1%, and in 21.4% of patients receiving PF bimatoprost 0.03%/timolol 0.5%.^101^ In a Phase III study, PF bimatoprost 0.01%/timolol 0.1% gel was compared to PF bimatoprost 0.03%/timolol 0.5% ophthalmic solution, showing non-inferior efficacy and a favourable safety profile.^102^ Indeed, no patient discontinued PF bimatoprost 0.01%/timolol 0.1% gel due to AEs, while a 2.9% discontinuation rate was reported with PF bimatoprost 0.03%/timolol 0.5% ophthalmic solution.^102^ Overall, these findings emphasize that innovative fixed combinations, particularly low-concentration gel formulations, can achieve effective IOP reduction with improved tolerability, thereby supporting adherence and addressing important needs in glaucoma management.

#### Fixed versus non-fixed combination

Fixed combination medications are a valuable option in glaucoma management, particularly when monotherapy failed to provide sufficient IOP reduction.^11^ Numerous studies supported the efficacy of various fixed combinations in IOP reductions.^100,103–106^ The first fixed combination of bimatoprost 0.03% and timolol 0.5% demonstrated non-inferior IOP-lowering efficacy than non-fixed combination and was particularly effective in patients insufficiently controlled on prostaglandin analogue monotherapy. Interestingly, the fixed combination had better safety profile than individual components alone.^107,108^ At three months, mean diurnal IOP decreases ranged from 8.1 mmHg with fixed combination to 6.4–7.9 mmHg with bimatoprost or timolol alone. A higher proportion of patients on fixed combination achieved significant IOP reductions (>20%) and target pressures (<18 mmHg) compared to bimatoprost and timolol.^107^ Importantly, fixed combination also showed a lower incidence of conjunctival hyperaemia than bimatoprost or non-fixed combinations, with rates ranging from 8.5% to 22.7%, compared to up to 38.5% in bimatoprost groups.^107,108^ The improved safety profile of the fixed combination compared to individual components may be attributed to the complementary mechanisms of action and pharmacological interactions between the drugs. Combining bimatoprost and timolol in a single formulation allows for effective IOP reduction at potentially lower individual drug concentrations, which might reduce the incidence of side effects such as conjunctival hyperaemia. Additionally, timolol's β-receptor antagonist properties could mitigate some of the vasodilatory effects of bimatoprost, leading to decreased ocular redness. Russ et al. have shown that short-term use (three months) of fixed combinations of prostaglandin analogues (latanoprost, bimatoprost, and travoprost) with 0.5% timolol maleate could still adversely affect the ocular surface.^109^ However, it is unsure if the treatments used in this study were preserved or not, and if this AE could be attributed to preservatives.^109^ Given these data, there is a need for careful patient monitoring when using non-fixed or fixed combinations.

### Clinical implications and therapeutic choices

#### Treatment adherence enhancement

PF glaucoma medications offer significant advantages in efficacy and patient experience, particularly for patients with OSD.^57,67,110^ Despite challenges such as higher costs (see Section “Optimization of health costs”), they are advantageous for many patients, particularly those at risk of surgical intervention.^47,78^ Switching to PF drops improved patient-reported outcomes, including quality of life, and sustained IOP control, making them ideal for personalized care, especially for patients with intolerance to preserved regimens.^42,48,53,57,69^ Low-concentration formulations can also reduce AEs and improve adherence,^111^ as seen with timolol 0.1%, which has a better safety profile and patient acceptance (measured through the COMTOL questionnaire^112^) compared to timolol 0.5%.^89,113^

On the other hand, fixed combinations could be beneficial for patients needing additional IOP-lowering therapy.^106,114–116^ By reducing administration frequency and dosing complexity, they improve adherence and overall patient experience.^117–120^ Studies confirmed higher adherence with fixed combination therapies, especially PF ones, compared to non-fixed or preserved combinations.^121–125^ Prostaglandin analogues/timolol fixed combinations showed better persistence and fewer discontinuations over time compared to separate agents.^126–132^ In a randomized clinical trial comparing two PF fixed combination of bimatoprost with timolol, overall treatment tolerability was higher with the gel formulation than with the eye drops, with 96–97% of patients and 96% of investigators reporting very satisfactory or satisfactory tolerability.^102^ This new gel formulation may represent a more favourable alternative to existing treatment options, offering potential improvements in both patient compliance and sustained drug delivery. Combining timolol with prostaglandin analogues in fixed combinations generally improves tolerability by reducing common ocular side effects.^133–135^ Fixed combinations also lower the risk of dosing errors, ensuring more consistent IOP management.^122^ Overall, available literature supports the concept that low-concentration PF fixed combinations provide a straightforward, reliable treatment option, helping to minimize treatment errors and improve adherence.

#### Quality-of-life improvement

In the context of treatment simplification approaches, improving patients’ quality of life goes beyond controlling IOP, since it involves minimizing treatment burden, AEs, and discomfort. While patients with early-stage glaucoma often retain good visual function, patients with advanced disease face significant declines in quality of life,^11^ deepened by the inconvenience of complex regimens, medication intolerance, and financial strain.^57^ Previous reports showed that switching to PF eye drops markedly improved treatment satisfaction^73,136,137^ and visual-related quality of life by reducing ocular surface irritation, the need for artificial tears, and drop discomfort.^76,138–141^ PF fixed combinations further enhance these benefits by simplifying regimens and limiting cumulative exposure to harmful agents such as BAK, which has been closely associated with OSD and reduced quality of life.^136,142^ These improvements extend beyond subjective tolerability and may translate into better adherence, fewer treatment disruptions, and more stable long-term outcomes.^48,140^ Thus, by reducing the toxic load and simplifying therapy, treatment simplification strategies support both clinical effectiveness and meaningful improvements in patients’ quality of life.

#### Sensitive populations

OSD is prevalent in glaucoma patients, especially due to long-term use of anti-glaucoma medications.^143–145^ Preservatives, particularly BAK, are strongly linked to OSD development and worsening, with symptoms increasing over time and with the number of medications used. Mylla Boso et al. demonstrated that each additional BAK-containing eyedrop was associated with a twofold increase in the odds of abnormal lissamine staining, a tool to assess OSD.^144^ Moreover, a recent survey found 45% of ophthalmologists see OSD in at least 25% of their patients, with redness (91.9%) and conjunctival hyperaemia (75.6%) as the most frequent symptoms.^146^ BAK was identified as the main cause of OSD by 90% of respondents. To manage OSD, physicians frequently switched to PF medications (33.7%) or drops with alternative preservatives (20.4%). Most respondents also prescribed artificial tears (84.6%) to alleviate symptoms.^146^ It is noteworthy that ocular surface treatment significantly improves visual acuity, OSDI scores, bulbar redness, and fluorescein staining, without having to stop IOP-lowering medication.^144^

Elderly patients are more prone to both ocular surface irritation and systemic AEs, including cardiovascular complications.^147^ Lower-concentration treatments, especially timolol, help avoid complications in this population of patients, who often face physical challenges that can alter proper medication use.^148^ As mentioned in Section “Treatment adherence enhancement”, fixed combination therapies further support adherence. This is particularly the case in elderly or patients with motor or cognitive impairments, by reducing both the number of required eye drops and the dosing regimen from twice to once daily. Once-daily combinations such as latanoprost/timolol were more manageable for patients with limited dexterity, poor vision, or memory issues.^149^ Simplifying regimens also helped prevent missed doses in patients taking multiple medications, which usually happens in elderly, supporting long-term disease control and easing caregiver responsibilities.^120,122^

## Beyond clinical benefits: Global impact of “Less is better”

Treatment simplification approaches in glaucoma management extend benefits beyond clinical outcomes. They may reduce the environmental impact, lower healthcare costs, and support more efficient prescription practices. This strategy could promote sustainability and enhance overall healthcare system efficiency.

### Reduction of environmental impact

#### Less pollution due to preservatives

The release of toxic agents such as BAK into the environment was reported to lead to aquatic toxicity and environmental persistence. Indeed, when used as a disinfectant, BAK has been frequently found in various environmental mediums such as wastewater influent and groundwater.^150^ Once in the environment, BAK can be lethal to aquatic organisms, disrupt local ecosystems and contribute to the broader issue of antimicrobial resistance.^150^ Although direct data linking ophthalmic BAK use to environmental harm are limited, these findings suggest that reducing overall BAK usage may help lower environmental exposure. Transitioning to PF formulations could significantly reduce this environmental impact, benefiting both ecosystems and human health.

#### Reduction of plastic and pharmaceutical waste

In addition to the environmental concerns related to preservatives, the plastic and pharmaceutical waste generated by glaucoma medication packaging is another area where improvements can be made. A study in intensive care settings has shown that medication waste, particularly with intravenous drugs such as anaesthetics used for surgeries, was both substantial (over 10%) and largely unavoidable due to clinical complexity.^151,152^ An anaesthesia study on surgery reported 45% of propofol waste, which could be reduced by using smaller vials, avoiding unnecessary drug disposal.^153^ While these findings originate from non-ophthalmic settings, they illustrate the broader challenge of pharmaceutical waste across healthcare systems and provide a conceptual framework relevant to chronic diseases such as glaucoma.

Fixed combinations decrease the number of vials required for treatment, leading to a reduction in plastic waste, packaging, and energy costs associated with production and transport. Ophthalmic studies have highlighted that minimizing the number of medications not only simplifies regimens,^154^ but may also align with sustainable healthcare practices by reducing the overall pharmaceutical burden on the environment.^155^

On the other hand, multi-dose vials can significantly reduce the overall amount of plastic waste generated by glaucoma medications. For patients with glaucoma, multi-dose delivery systems have been proposed as a way to reduce plastic waste, and some evidence suggests they may also support adherence by simplifying administration compared to single-use containers.^156^ These systems not only simplify the medication administration process but also provide an environmentally sustainable alternative to the traditional single-use plastic containers. However, despite these advances, certain innovative formulations, such as gel-based preparations, are not yet compatible with PF multi-dose bottles, and thus still require single-dose packaging to deliver their therapeutic effects.

#### Less carbon footprint with reduced medical travel

Multiple studies have shown that fixed combinations and PF formulations improve adherence, enhance tolerability, and reduce AEs (see Section “Changing The Paradigm”). As a result, patients on these better-tolerated therapies could be less likely to require additional clinic visits related to side effects or the need of therapy modification. A recent report identified patient and staff travel as the primary source of emissions within glaucoma care pathways.^157^ Although a direct causal link between specific glaucoma therapies and carbon footprint reduction has not yet been formally established, improved treatment stability could potentially allow for longer follow-up intervals in selected patients. This approach may help maintain high-quality care while reducing the number of patient journeys, an especially relevant benefit in rural or underserved areas where access to ophthalmologists may involve long travel distances.

### Optimization of health costs

#### Reduction of medical visits and supportive treatments

Previous studies have demonstrated that fixed combinations can improve patient adherence (see Section “Sensitive populations”), reduce the likelihood of medication-related complications, and may be associated with a lower frequency of follow-up visits^128,158^ and improved quality of life. Fixed combinations were described as cost-effective treatment option for patients with glaucoma.^156,159^ Brand-name combination medications were often less expensive than purchasing their individual components separately, further strengthening their cost-effectiveness.^160^ In addition, improved adherence resulted in reduced overall costs over time. For example, switching from bimatoprost 0.03% to bimatoprost 0.01% led to cost savings after the initial years of treatment, with a lifetime reduction reaching US$3433.^161^

Furthermore, because preserved IOP-lowering medications are commonly linked to ocular surface disorders (see Section “Impact on the ocular surface and deeper tissues”), using PF therapies may reduce the need for additional treatments, including artificial tears.^20^ In the UK, prescription costs for PF treatments increased significantly in recent years, but their benefits over the preserved alternatives could justify this rise, especially as patients experienced fewer complications and required less supplemental care.^162^

#### Surgeries and their complications

A significant portion of healthcare expenditures in glaucoma management arises from surgical interventions, such as trabeculectomies and cataract surgeries, along with their associated complications. It has been demonstrated that optimizing medical therapy through the use of less concentrated, PF, and fixed combination eye drops can help control disease progression and delay or avoid surgical interventions.^163^ Fewer surgeries would be expected to translate into fewer complications, reduced hospitalization, and lower expenditures on postoperative care and rehabilitation, factors particularly relevant in managing elderly patients and those with multiple health conditions.

## Challenges and perspectives

The increasing focus on innovative glaucoma treatment strategies reflects a growing effort to improve patient adherence, comfort, and overall disease management. The higher cost of PF medications, driven by more complex manufacturing and packaging processes, remains a key factor influencing their wider adoption. This price gap between preserved and PF medications^164^ has practical implications for healthcare systems and patients, often limiting access despite the known benefits of PF treatments in improving ocular surface health and treatment adherence.

In addition to cost, treatment optimization remains an ongoing concern. While fixed combination therapies reduce dosing complexity and can improve adherence, they inherently limit the flexibility to individually adjust medication components or timing. For some patients, particularly those with variable responses or specific tolerance issues, this lack of flexibility can delay optimal IOP control.^60^ Using separate agents could allow therapy to be escalated only when necessary, supporting a stepwise, individualized approach that balances efficacy with the goal of limiting treatment burden. Furthermore, although combinations involving prostaglandin analogues are commonly used and generally effective and well tolerated, no single combination has proven universally ideal.^165^ There is also continued debate over whether fixed combinations consistently outperform individually dosed regimens in terms of efficacy.^8^

Treatment simplification in glaucoma management is not limited to topical formulations alone. Selective Laser Trabeculoplasty (SLT) offers a cost-effective, non-invasive alternative that can be used as a first-line treatment.^11,166^ By enhancing aqueous outflow, SLT effectively lowers IOP and may reduce or eliminate the need for daily topical medications, improving adherence and reducing the risk of medication-related AEs.^166,167^ This treatment could offer a convenient alternative that supports long-term ocular health while reducing dependence on eyedrops. This not only simplifies disease management but may also contribute to long-term cost savings. Systemic treatments such as oral acetazolamide may also play a role in patients needing a rapid IOP reduction or when topical options are insufficient or poorly tolerated.^168^ However, their long-term use is limited by systemic side effects. Beyond established therapies, emerging treatment modalities such as implants may be considered when adherence, treatment burden, and tolerability issues need to be addressed.

Another important barrier to broader use of PF and fixed combination therapies is limited physician awareness regarding their benefits. Many clinicians, particularly general practitioners who issue most prescriptions, may underestimate the toxic effects of common preservatives such as BAK on the ocular surface and patient comfort. National guidelines can further influence these trends. For instance, the Danish Guidelines restrict substitution with generics and explicitly encourage the use of PF formulations, illustrating how structured recommendations may help align prescribing practices with patient-centered outcomes.^169^ These discrepancies underscore the urgent need for enhanced education and standardized prescribing guidelines to improve understanding of PF formulations and fixed combinations, ultimately optimizing patient outcomes.

Importantly, treatment simplification should also be applied to clinical decision-making and care delivery. Education of practitioners should be strengthened to support appropriate adaptation of treatment over time. Risk-stratified follow-up schedules and intentional de-escalation of therapy in stable disease may help avoid overtreatment while preserving visual function. However, it is important to note that minimalism does not imply undertreatment but rather refers to selecting the least burdensome regimen that achieves the patient's target IOP, with timely escalation to additional medical, laser, or surgical interventions when clinically indicated, particularly in advanced disease. New monitoring tools, including AI-based approaches for detecting glaucomatous progression, may further support individualized treatment decisions by identifying disease worsening earlier and more reliably.^170^ This would contribute to treatment simplification in glaucoma care with the reduction of unnecessary testing and clinic visits.

Finally, a need for robust long-term evidence persists. While current data support short- and mid-term benefits such as better adherence, fewer AEs, and improved ocular surface outcomes (see Section “Changing the paradigm”), the long-term impact of these therapies on IOP control, disease progression, and visual outcomes remains insufficiently studied. Given the lifelong nature of glaucoma, future research must assess the durability of these benefits over time, as well as their cost-effectiveness and influence on quality of life.

PF fixed combination low-concentration therapies strongly support the goals of modern glaucoma management and successfully integrating them into routine care will be achieved by addressing economic, clinical, and evidence-based opportunities.

## Conclusion

Treatment simplification approaches in glaucoma care allow a “Less is better” concept and promote simpler, safer, and more sustainable treatment strategies without compromising efficacy. By favouring PF and low-concentration formulations, it reduces AEs and improves patient comfort and adherence. Fixed combinations further streamline regimens, minimizing dosing errors and enhancing long-term IOP control, leading to better clinical outcomes. Environmentally, it reduces preservative contamination and plastic waste, while economically, it could lower long-term healthcare costs. Overall, this strategy supports a more patient-centred, efficient, and environmentally responsible model of glaucoma management.
